# Polymorphisms in endoplasmic reticulum aminopeptidase genes are associated with cervical cancer risk in a Chinese Han population

**DOI:** 10.1186/s12885-020-06832-2

**Published:** 2020-04-22

**Authors:** Chuanyin Li, Yaheng Li, Zhiling Yan, Shuying Dai, Shuyuan Liu, Xia Wang, Jun Wang, Xinwen Zhang, Li Shi, Yufeng Yao

**Affiliations:** 1 Institute of Medical Biology, Chinese Academy of Medical Sciences & Peking Union Medical College, Kunming, 650118 China; 2 Department of Gynaecologic Oncology, The 3rd Affiliated Hospital of Kunming Medical University, Kunming, 650118 China; 3 School of Basic Medical Science, Kunming Medical University, Kunming, 650500 China

**Keywords:** Endoplasmic reticulum aminopeptidase, Single nucleotide polymorphisms, Association, Susceptibility, Cervical intraepithelial neoplasia, Cervical cancer

## Abstract

**Background:**

Antigen-processing machinery molecules play crucial roles in infectious diseases and cancers. Studies have shown that polymorphisms in endoplasmic reticulum aminopeptidase (ERAP) genes can influence the enzymatic activity of ERAP proteins and are associated with the risk of diseases. In the current study, we evaluated the influence of ERAP gene (*ERAP1* and *ERAP2*) polymorphisms on susceptibility to cervical intraepithelial neoplasia (CIN) and cervical cancer.

**Methods:**

Six single nucleotide polymorphisms (SNPs) in *ERAP1* and 5 SNPs in *ERAP2* were selected and genotyped in 556 CIN patients, 1072 cervical cancer patients, and 1262 healthy control individuals. Candidate SNPs were genotyped using SNaPshot assay. And the association of these SNPs with CIN and cervical cancer was analysed.

**Results:**

The results showed that allelic and genotypic frequencies of rs26653 in *ERAP1* were significantly different between cervical cancer and control groups (*P* = 0.001 and 0.004). The allelic frequencies of rs27044 in *ERAP1* and rs2287988 in *ERAP2* were significantly different between control and cervical cancer groups (*P* = 0.003 and 0.004). Inheritance model analysis showed that genotypes of rs27044, rs26618, rs26653 and rs2287988 SNPs may be associated with the risk of cervical cancer (*P* = 0.003, 0.004, 0.001 and 0.002). Additionally, haplotype analysis results showed that the *ERAP1* haplotype, rs27044C-rs30187T-rs26618T-rs26653G-rs3734016C, was associated with a lower risk of cervical cancer (*P* = 0.001). The *ERAP2* haplotypes rs2549782G- rs2548538A-rs2248374A-rs2287988G-rs1056893T (*P* = 0.009 and 0.006) and rs2549782T-rs2548538T-rs2248374G-rs2287988A-rs1056893T (*P* = 0.003 and 0.009) might be associated with cervical cancer and the development from CIN to cervical cancer.

**Conclusion:**

Our results indicated that rs27044, rs26618 and rs26653 in *ERAP1* and rs2287988 in *ERAP2* influenced susceptibility to cervical cancer.

## Background

The antigen-processing machinery (APM) is composed of the proteasome, where exogenous and tumour antigens are degraded into peptides; transporters associated with antigen presentation (TAPs), which are responsible for the translocation of peptide precursors; endoplasmic reticulum aminopeptidases (ERAPs), which trim the peptides to fit major histocompatibility complex (MHC) molecules; and MHC proteins, which present antigen peptides on the cell surface [1, 2]. Human ERAPs, which belong to the oxytocinase subfamily of M1 metalloproteases, are crucial molecules of the APM. In the endoplasmic reticulum lumen, ERAP1 and ERAP2 trim peptides into their final length to render them suitable for loading onto HLA class I molecules [3, 4]. Recently, several studies have shown that ERAP proteins play crucial roles in autoimmune diseases [5, 6], infectious diseases [7, 8], and cancers [9, 10].

Cervical cancer is the fourth most common malignancy in women globally [11]. Persistent human papillomavirus (HPV) infection confers a high risk of cervical cancer [12, 13]. Since the HLA class I antigen-presenting system is responsible for the presentation of foreign and cancerous antigens to the immune system [14, 15], and ERAPs downregulation was observed in cervical cancer [16, 17], therefore, ERAP proteins may play crucial roles in the initiation and development of cervical cancer [18].

Previous studies revealed the association between single nucleotide polymorphisms (SNPs) in ERAP genes (such as rs26653, rs30187, rs27044, rs2549782, rs2287988, rs26618, rs3734016, rs27037, rs2248374, rs2548538 and rs1056893) and autoimmune and infectious diseases [19–22], and human cancers [23–26]. Moreover, functional studies have shown that SNPs in ERAP genes could affect the enzymatic activity and selectivity of ERAP proteins (such as rs27044, and rs30187 in *ERAP1* gene, rs2287988 in *ERAP2* gene) [27–32], and affect the conformation of ERAP proteins (such as rs27044, rs30187 and rs26653 in *ERAP1* gene; rs2549782 and rs2287988 in *ERAP2* gene) [31, 33, 34]. These results suggested that SNPs in ERAP genes could be valuable to be selected for association studies. Thus, in the current study, we selected 11 SNPs located in *ERAP1* (rs27037, rs27044, rs30187, rs26618, rs26653 and rs3734016) and *ERAP2* (rs2549782, rs2548538, rs2248374, 2,287,988 and rs1056893) and investigated their distribution in patients with cervical intraepithelial neoplasia (CIN) and cervical cancer and healthy individuals, to assess their association with the initiation and development of cervical cancer.

## Methods

### Study population

In the current study, a total of 556 patients with CIN and 1072 patients with cervical cancer were enrolled at the Third Affiliated Hospital of Kunming Medical University from May 2014 to August 2018. The inclusion criteria were as follows: 1) diagnosis of CIN or cervical cancer according to *Current Diagnosis and Treatment: Obstetrics and Gynaecology* and International Federation of Gynaecology and Obstetrics (2009) guidelines; 2) no other malignancy in patients and no history of cancer or other chronic diseases in control individuals; and 3) no preoperative neoadjuvant therapies (including chemotherapy and radiotherapy). The exclusion criteria for patients were as follows: 1) a prior history of primary cancer other than cervical cancer; 2) malignant tumours other than cervical cancer; 3) currently receiving radiotherapy or chemotherapy; and 4) an unclear diagnosis. Over the same period, 1262 healthy women from a health screening project at the same hospital were enrolled as controls.

### SNP selection and genotyping

Six SNPs located in *ERAP1* and 5 SNPs located in *ERAP2* were selected in the current study. The minor allele frequency should be over 0.05 in East Asian population (http://asia.ensembl.org/index.html). The details of the selected SNPs are displayed in Supplementary Table 1. Venous blood samples were collected for the extraction of genomic DNA, using the QIAamp Blood Mini Kit (Qiagen NV, Venlo, Netherlands). Genotyping of the 11 SNPs was performed using the SNaPshot SNP assay (Thermo Fisher Scientific, Waltham, MA, USA), and results were analysed using GeneMapper TM 4.0 software (Applied Biosystems, Foster City, CA, USA). For quality control, 5% of samples from the case and control groups were genotyped twice with unique analysis serial numbers and the reproducibility was found to be 100%.

### Statistical analysis

Hardy-Weinberg equilibrium (HWE) was evaluated to determine the representativeness of the study population. The differences in age among the CIN, cervical cancer, and control groups were analysed using a one-way ANOVA, with a least significant difference test for multiple comparison correction. Allelic and genotypic frequencies of these SNPs were compared between different groups using a Chi-square test and odds ratios (ORs) with associated 95% confidence intervals (CIs) were calculated. Additionally, linkage disequilibrium (LD) was calculated and a *D’* value greater than 0.80 was considered to indicate LD. The haplotypes among these SNPs were analysed using SHEsis software [35, 36]. Subsequently, the distribution of the haplotypes between different groups was compared using a Chi-square test. In addition, inheritance analysis adjusted by age was performed using SNPstats software to identify the relationship between genotypes at these SNPs and cervical cancer [37]. In the inheritance analysis, four inheritance models (codominant, dominant, recessive, and log-additive) were analysed. Simultaneously, Akaike information criterion (AIC) and Bayesian information criterion (BIC) values were calculated to determine the inheritance model with the best fit, i.e. the model with the smallest AIC and BIC values [37]. The statistical power was calculated using Power and Sample Size software (V3.1.2) [38]. Bonferroni correction was performed for multiple comparisons, after which the statistical significance threshold was set at *P* < 0.0045 (0.05/11).

## Results

### Characteristics of the subjects

Table 1 shows the clinical data of the subjects in the present study. There was no significant difference in age among the control, CIN, and cervical cancer groups (*P* > 0.05, *F* = 1.438), as evaluated by one-way ANOVA. In the CIN group, there were 65 patients with low-grade CIN (I/II) and 491 patients with high-grade CIN (III). In the cervical cancer group, there were 151 patients with adenocarcinoma, 903 patients with squamous cell carcinoma, and 18 patients with other pathological types.

**Table 1:** Characteristics of the subjects enrolled in the current study

	Cervical cancer	CIN	Control	***F***	***P***-value
**N**	1072	556	1262		
**Age**	47.81 ± 10.21	47.42 ± 9.37	48.28 ± 9.60	1.438	0.238
**Pathological types**					
SCC	903				
AC	151				
Others	18				
**Stages of CIN**					
Low degrade of CIN (I/II)		65			
High Degrade of CIN (III)		491			

**Note:***SCC* squamous cell carcinoma; AC, Adenocarcinoma

### Association of the eleven SNPs with CIN and cervical cancer

All 11 SNPs were in HWE in the control group (*P* > 0.05) (Supplementary Table 1). The allelic and genotypic frequencies of these SNPs are presented in Tables 2 and 3. The results showed that the allelic and genotypic frequencies of rs26618 (*P* = 0.021 and 0.016, respectively), rs26653 (*P* = 0.001 and 0.004), rs27044 (*P* = 0.003 and 0.012) and rs30187 (*P* = 0.008 and 0.020) in *ERAP1* (Table 2) and rs2248374 (*P* = 0.014 and 0.020) and rs2287988 (*P* = 0.004 and 0.007) in *ERAP2* (Table 3) were significantly different between cervical cancer and control groups. Additionally, the allelic and genotypic distributions of rs2248374 (*P* = 0.015 and 0.041, respectively) and rs2287988 (*P* = 0.014 and 0.039) in *ERAP2* were significantly different between CIN and cervical cancer groups (Table 3). However, after Bonferroni correction, only rs26653, rs27044, and rs2287988 were associated with cervical cancer risk (*P* < 0.0045). The results indicated that, in *ERAP1*, the G allele of rs26653 may be associated with a lower risk of cervical cancer compared with C allele (OR = 0.829; 95% CI: 0.738–0.930) and the C allele of rs27044 may be a protective factor for cervical cancer (OR = 0.838, 95% CI: 0.746–0.941). Moreover, the G allele of rs2287988 in *ERAP2* may be associated with a higher risk of cervical cancer (OR = 1.187, 95% CI: 1.057–1.332). There were no SNPs in *ERAP1* (Table 2) or *ERAP2* (Table 3) that exhibited a significantly different distribution between the CIN and control groups or between the CIN and cervical cancer groups after Bonferroni correction (*P* > 0.0045).

**Table 2:** The allelic and genotypic distribution among control, CIN and cervical cancer groups of SNPs in *ERAP1* gene

SNPs	Control (Freq)	CIN (Freq)	Cervical cancer (Freq)	Cervical cancer vs Control			CIN vs Control		Cervical cancer vs CIN
				*P*-value	OR[95%CI]	*P-*value	OR[95%CI]	*P*-value	OR[95%CI]
rs27037									
G	1372 (54.4%)	592 (53.2%)	1092 (50.9%)	0.020	1.147 [1.022–1.288]	0.532	1.046 [0.908–1.205]	0.212	1.097 [0.949–1.268]
T	1152 (45.6%)	520 (46.8%)	1052 (49.1%)						
G/G	359 (28.4%)	161 (29.0%)	283 (26.4%)	0.020		0.323		0.462	
G/T	654 (51.8%)	270 (48.6%)	526 (49.1%)						
T/T	249 (19.8%)	125 (22.5%)	263 (24.5%)						
rs27044									
G	1350 (53.5%)	611 (54.9%)	1240 (57.8%)	0.003	0.838 [0.746–0.941]	0.416	0.943 [0.818–1.086]	0.114	0.889 [0.768–1.029]
C	1174 (46.5%)	501 (45.1%)	904 (42.2%)						
G/G	362 (28.7%)	175 (31.5%)	360 (33.6%)	0.012		0.454		0.196	
G/C	626 (49.6%)	261 (46.9%)	520 (48.5%)						
C/C	274 (21.7%)	120 (21.6%)	192 (17.9%)						
rs30187									
C	1318 (52.2%)	589 (53.0%)	1203 (56.1%)	0.008	0.855 [0.761–0.960]	0.678	0.970 [0.843–1.118]	0.087	0.881 [0.762–1.019]
T	1206 (47.8%)	523 (47.0%)	941 (43.9%)						
C/C	343 (27.2%)	169 (30.4%)	347 (32.4%)	0.020		0.151		0.134	
C/T	632 (50.0%)	251 (45.1%)	509 (47.5%)						
T/T	287 (22.8%)	136 (24.5%)	216 (20.1%)						
rs26618									
T	1852 (73.4%)	785 (70.6%)	1508 (70.3%)	0.021	1.162 [1.023–1.321]	0.083	1.148 [0.982–1.342]	0.879	1.012 [0.863–1.187]
C	672 (26.6%)	327 (29.4%)	636 (29.7%)						
T/T	678 (53.7%)	285 (51.3%)	546 (50.9%)	0.016		0.076		0.989	
C/T	496 (39.3%)	215 (38.7%)	416 (38.8%)						
C/C	88 (7.0%)	56 (10.1%)	110 (10.3%)						
rs26653									
C	1227 (48.6%)	574 (51.6%)	1143 (53.3%)	0.001	0.829 [0.738–0.930]	0.095	0.887 [0.770–1.021]	0.359	0.934 [0.808–1.080]
G	1297 (51.4%)	538 (48.4%)	1001 (46.7%)						
C/C	281 (22.3%)	142 (25.5%)	299 (27.9%)	0.004		0.224		0.591	
G/C	665 (52.7%)	290 (52.2%)	545 (50.8%)						
G/G	316 (25.0%)	124 (22.3%)	228 (21.3%)						
rs3734016									
C	2159 (85.5%)	947 (85.2%)	1801 (84.0%)	0.145	0.888 [0.756–1.041]	0.767	0.970 [0.795–1.184]	0.387	0.915 [0.748–1.119]
T	365 (14.5%)	165 (14.8%)	343 (16.0%)						
C/C	921 (73.0%)	404 (72.7%)	752 (70.1%)	0.318		0.832		0.500	
C/T	317 (25.1%)	139 (25.0%)	297 (27.7%)						
T/T	24 (1.9%)	13 (2.3%)	23 (2.1%)						

**Note:** The statistical significant threshold was set at *P* < 0.0045 after Bonferroni correction

**Table 3:** The allelic and genotypic distribution among control, CIN and cervical cancer groups of SNPs in *ERAP2* gene

SNPs	Control (Freq)	CIN (Freq)	Cervical cancer (Freq)	Cervical cancer vs Control			CIN vs Control		Cervical cancer vs CIN
				***P***-value	OR[95%CI]	***P***-value	OR[95%CI]	***P***-value	OR[95%CI]
rs2549782									
T	1418 (56.2%)	628 (56.5%)	1146 (53.5%)	0.062	1.117 [0.995–1.253]	0.869	0.988 [0.857–1.139]	0.100	1.130 [0.977–1.307]
G	1106 (43.8%)	484 (43.5%)	998 (46.5%)						
T/T	395 (31.3%)	173 (31.1%)	291 (27.1%)	0.089		0.906		0.214	
G/T	628 (49.8%)	282 (50.7%)	564 (52.6%)						
G/G	239 (18.9%)	101 (18.2%)	217 (20.2%)						
rs2548538									
T	1461 (57.9%)	638 (57.4%)	1185 (55.3%)	0.073	1.112 [0.990–1.249]	0.774	1.021 [0.885–1.178]	0.252	1.089 [0.941–1.261]
A	1063 (42.1%)	474 (42.6%)	959 (44.7%)						
T/T	439 (34.8%)	189 (34.0%)	337 (31.4%)	0.197		0.948		0.524	
A/T	583 (46.2%)	260 (46.8%)	511 (47.7%)						
A/A	240 (19.0%)	107 (19.2%)	224 (20.9%)						
rs2248374									
G	1396 (55.3%)	625 (56.2%)	1109 (51.7%)	0.014	1.155 [1.029–1.296]	0.616	0.964 [0.837–1.116]	0.015	1.198 [1.035–1.386]
A	1128 (44.7%)	487 (43.8%)	1035 (48.3%)						
G/G	382 (30.3%)	169 (30.4%)	269 (25.1%)	0.020		0.690		0.041	
A/G	632 (50.0%)	287 (51.6%)	571 (53.3%)						
A/A	248 (19.7%)	100 (18.0%)	232 (21.6%)						
rs2287988									
A	1407 (55.7%)	623 (56.0%)	1104 (51.5%)	0.004	1.187 [1.057–1.332]	0.875	0.989 [0.858–1.140]	0.014	1.200 [1.038–1.388]
G	1117 (44.3%)	489 (44.0%)	1040 (48.5%)						
A/A	387 (30.7%)	167 (30.0%)	267 (24.9%)	0.007		0.743		0.039	
A/G	633 (50.1%)	289 (52.0%)	570 (53.2%)						
G/G	242 (19.2%)	100 (18.0%)	235 (21.9%)						
rs1056983									
T	1462 (57.9%)	647 (58.2%)	1225 (57.1%)	0.587	1.033 [0.919–1.160]	0.884	0.989 [0.858–1.141]	0.567	1.044 [0.901–1.209]
C	1062 (42.1%)	465 (41.8%)	919 (42.9%)						
T/T	439 (34.8%)	195 (35.1%)	360 (33.6%)	0.830		0.990		0.831	
C/T	584 (46.3%)	257 (46.2%)	505 (47.1%)						
C/C	239 (18.9%)	104 (18.7%)	207 (19.3%)						

**Note:** The statistical significant threshold was set at *P* < 0.0045 after Bonferroni correction

### Inheritance model analysis

To evaluate the genotypic association of the 11 SNPs with CIN and cervical cancer, inheritance analysis was performed among cervical cancer, CIN, and control groups (Table 4, Table 5, and Supplementary Tables 2–5). The CC genotype of rs26618 was a risk factor for cervical cancer, compared with TT-CT genotype (*P* = 0.004; OR = 1.53, 95%CI: 1.14–2.05) in the recessive model (the best-fit inheritance model for the comparison between control and cervical cancer groups) (Table 4). The 2GG + CG genotype of rs26653 was associated with a lower risk of cervical cancer compared with the CC genotype (*P* = 0.001, OR = 0.82; 95% CI: 0.73–0.93) in the log-additive model (the best-fit inheritance model for the comparison between control and cervical cancer groups) (Table 4). The 2CC + CG genotype of rs27044 may be a protective factor against cervical cancer compared with the GG genotype (*P* = 0.003, OR = 0.84; 95% CI: 0.75–0.94) in the log-additive model (the best-fit inheritance model for the comparison between control and cervical cancer groups) (Table 4) and the GG-GA genotype of rs2287988 may be a risk factor for cervical cancer compared with the AA genotype (*P* = 0.002, *OR* = 1.33; 95% CI: 1.11–1.60) in the dominant model (the best fit inheritance model for the comparison between control and cervical cancer groups) (Table 5).

**Table 4:** Inheritance model analysis of SNPs in *ERAP1* gene between control and cervical cancer groups

SNPs	Models	Genotypes	Control (Freq)	Cervical cancer (Freq)	OR[95%CI]	*P*-value	AIC	BIC
rs27037	Condominant	G/G	359 (28.4%)	283 (26.4%)	1	0.020	3218.2	3241.2
		G/T	654 (51.8%)	526 (49.1%)	1.02 (0.84–1.23)			
		T/T	249 (19.8%)	263 (24.5%)	1.34 (1.06–1.69)			
	Dominant	G/G	359 (28.4%)	283 (26.4%)	1	0.280	3222.8	3240.1
		G/T-T/T	903 (71.6%)	789 (73.6%)	1.11 (0.92–1.33)			
	Recessive	G/G-G/T	1013 (80.2%)	809 (75.5%)	1	0.006	3216.2	3233.5
		T/T	249 (19.8%)	263 (24.5%)	1.32 (1.09–1.61)			
	Log-additive	–	–	–	1.15 (1.02–1.29)	0.020	3218.6	3235.8
rs27044	Condominant	G/G	362 (28.7%)	360 (33.6%)	1	0.012	3217.2	3240.2
		G/C	626 (49.6%)	520 (48.5%)	0.84 (0.70–1.01)			
		C/C	274 (21.7%)	192 (17.9%)	0.71 (0.56–0.89)			
	Dominant	G/G	362 (28.7%)	360 (33.6%)	1	0.012	3217.6	3234.9
		G/C-C/C	900 (71.3%)	712 (66.4%)	0.80 (0.67–0.95)			
	Recessive	G/G-G/C	988 (78.3%)	880 (82.1%)	1	0.021	3218.7	3235.9
		C/C	274 (21.7%)	192 (17.9%)	0.79 (0.64–0.97)			
	Log-additive	–	–	–	0.84 (0.75–0.94)	0.003	3215.2	3232.5
rs30187	Condominant	C/C	343 (27.2%)	347 (32.4%)	1	0.020	3218.2	3241.2
		C/T	632 (50.0%)	509 (47.5%)	0.80 (0.66–0.96)			
		T/T	287 (22.8%)	216 (20.1%)	0.74 (0.59–0.94)			
	Dominant	C/C	343 (27.2%)	347 (32.4%)	1	0.007	3216.6	3233.9
		C/T-T/T	919 (72.8%)	725 (67.6%)	0.78 (0.65–0.93)			
	Recessive	C/C-C/T	975 (77.2%)	856 (79.8%)	1	0.120	3221.6	3238.9
		T/T	287 (22.8%)	216 (20.1%)	0.86 (0.70–1.04)			
	Log-additive	–	–	–	0.86 (0.76–0.96)	0.009	3217.1	3234.3
rs26618	Condominant	T/T	678 (53.7%)	546 (50.9%)	1	0.016	3217.7	3240.7
		C/T	496 (39.3%)	416 (38.8%)	1.04 [0.88–1.24]			
		C/C	88 (7.0%)	110 (10.3%)	1.55 [1.15–2.10]			
	Dominant	T/T	678 (53.7%)	546 (50.9%)	1	0.180	3222.2	3239.5
		C/T-C/C	584 (46.3%)	526 (49.1%)	1.12 [0.95–1.32]			
	Recessive	T/T-C/T	1174 (93.0%)	962 (89.7%)	1	0.004	3215.9	3233.1
		C/C	88 (7.0%)	110 (10.3%)	1.53 [1.14–2.05]			
	Log-additive	–	–	–	1.16 [1.02–1.31]	0.023	3218.9	3236.1
rs26653	Condominant	C/C	281 (22.3%)	299 (27.9%)	1	0.004	3214.8	3237.8
		C/G	665 (52.7%)	545 (50.8%)	0.77 [0.63–0.94]			
		G/G	316 (25.0%)	228 (21.3%)	0.68 [0.54–0.86]			
	Dominant	C/C	281 (22.3%)	299 (27.9%)	1	0.002	3214.2	3231.5
		C/G-G/G	981 (77.7%)	773 (72.1%)	0.74 [0.61–0.89]			
	Recessive	C/C-C/G	946 (75.0%)	844 (78.7%)	1	0.034	3219.5	3236.7
		G/G	316 (25.0%)	228 (21.3%)	0.81 [0.67–0.98]			
	Log-additive	–	–	–	0.82 [0.73–0.93]	0.001	3213.5	3230.7
rs3734016	Condominant	C/C	921 (73.0%)	752 (70.2%)	1	0.330	3223.8	3246.8
		C/T	317 (25.1%)	297 (27.7%)	1.15 (0.95–1.38)			
		T/T	24 (1.9%)	23 (2.1%)	1.17 (0.65–2.09)			
	Dominant	C/C	921 (73.0%)	752 (70.2%)	1	0.140	3221.8	3239.0
		C/T-T/T	341 (27.0%)	320 (29.9%)	1.15 (0.96–1.37)			
	Recessive	C/C-C/T	1238 (98.1%)	1049 (97.8%)	1	0.690	3223.8	3241.1
		T/T	24 (1.9%)	23 (2.1%)	1.13 (0.63–2.01)			
	Log-additive	–	–	–	1.13 (0.96–1.33)	0.150	3221.9	3239.1

**Note:** The statistical significant threshold was set at *P* < 0.0045 after Bonferroni correction

**Table 5:** Inheritance model analysis of SNPs in *ERAP2* gene between control and cervical cancer groups

SNPs	Models	Genotypes	Control (Freq)	Cervical cancer (Freq)	OR[95%CI]	*P*-value	AIC	BIC
rs2549782	Codominant	T/T	395 (31.3%)	291 (27.1%)	1	0.088	3221.1	3244.2
		G/T	628 (49.8%)	564 (52.6%)	1.22 (1.01–1.47)			
		G/G	239 (18.9%)	217 (20.2%)	1.23 (0.97–1.56)			
	Dominant	T/T	395 (31.3%)	291 (27.1%)	1	0.028	3219.2	3236.4
		G/T-G/G	867 (68.7%)	781 (72.8%)	1.22 (1.02–1.46)			
	Recessive	T/T-G/T	1023 (81.0%)	855 (79.8%)	1	0.430	3223.4	3240.6
		G/G	239 (18.9%)	217 (20.2%)	1.09 (0.89–1.33)			
	Log-additive	–	–	–	1.12 (1.00–1.26)	0.057	3220.4	3237.6
rs2548538	Condominant	T/T	439 (34.8%)	337 (31.4%)	1	0.200	3222.8	3245.8
		A/T	583 (46.2%)	511 (47.7%)	1.14 (0.95–1.37)			
		A/A	240 (19.0%)	224 (20.9%)	1.22 (0.96–1.53)			
	Dominant	T/T	439 (34.8%)	337 (31.4%)	1	0.089	3221.1	3238.4
		A/T-A/A	823 (65.2%)	735 (68.6%)	1.16 (0.98–1.38)			
	Recessive	T/T-A/T	1022 (81.0%)	848 (79.1%)	1	0.260	3222.7	3240.0
		A/A	240 (19.0%)	224 (20.9%)	1.12 (0.92–1.38)			
	Log-additive	–	–	–	1.11 (0.99–1.24)	0.080	3220.9	3238.2
rs2248374	Condominant	G/G	382 (30.3%)	269 (25.1%)	1	0.020	3218.1	3241.2
		A/G	632 (50.0%)	571 (53.3%)	1.28 (1.06–1.56)			
		A/A	248 (19.7%)	232 (21.6%)	1.33 (1.05–1.69)			
	Dominant	G/G	382 (30.3%)	269 (25.1%)	1	0.005	3216.2	3233.5
		A/G-A/A	880 (69.7%)	803 (74.9%)	1.30 (1.08–1.56)			
	Recessive	G/G-A/G	1014 (80.3%)	840 (78.4%)	1	0.230	3222.6	3239.8
		A/A	248 (19.7%)	232 (21.6%)	1.13 (0.92–1.38)			
	Log-additive	–	–	–	1.16 (1.03–1.31)	0.012	3217.7	3235.0
rs2287988	Codominant	A/A	387 (30.7%)	267 (24.9%)	1	0.007	3216.0	3239.0
		A/G	633 (50.1%)	570 (53.2%)	1.30 (1.07–1.58)			
		G/G	242 (19.2%)	235 (21.9%)	1.41 (1.11–1.78)			
	Dominant	A/A	387 (30.7%)	267 (24.9%)	1	0.002	3214.5	3231.8
		A/G-G/G	875 (69.3%)	805 (75.1%)	1.33 (1.11–1.60)			
	Recessive	A/A-A/G	1020 (80.8%)	837 (78.1%)	1	0.100	3221.3	3238.6
		G/G	242 (19.2%)	235 (21.9%)	1.18 (0.97–1.45)			
	Log-additive	–	–	–	1.19 (1.06–1.34)	0.003	3215.3	3232.5
rs1056983	Condominant	T/T	439 (34.8%)	360 (33.6%)	1	0.820	3225.6	3248.6
		C/T	584 (46.3%)	505 (47.1%)	1.06 (0.88–1.27)			
		C/C	239 (18.9%)	207 (19.3%)	1.06 (0.84–1.34)			
	Dominant	T/T	439 (34.8%)	360 (33.6%)	1	0.530	3223.6	3240.9
		C/T-C/C	823 (65.2%)	712 (66.4%)	1.06 (0.89–1.25)			
	Recessive	T/T-C/T	1023 (81.0%)	865 (80.7%)	1	0.810	3223.9	3241.2
		C/C	239 (18.9%)	207 (19.3%)	1.03 (0.83–1.26)			
	Log-additive	–	–	–	1.03 (0.92–1.16)	0.580	3223.7	3241.0

**Note:** The statistical significant threshold was set at *P* < 0.0045 after Bonferroni correction

### Linkage disequilibrium (LD) and haplotype analysis of SNPs in *ERAP1* and *ERAP2*

The results of LD analysis showed that rs26618, rs26653, rs27044, rs30187, and rs3734016 in *ERAP1* and rs2248374, rs2549782, rs2287988, rs2548538, and rs1056893 in *ERAP2* were in LD (*D’* > 0.80) (Supplementary Tables 6, 7). Subsequently, we constructed the haplotypes, rs27044-rs30187-rs26618-rs26653-rs3734016 and rs2549782-rs2548538-rs2248374-rs2287988-rs1056893. The distribution of these haplotypes (with a frequency of more than 3%) was compared in a pairwise manner among the cervical cancer, CIN, and control groups (Tables 6 and 7). The *ERAP1* haplotype, rs27044C-rs30187T-rs26618T-rs26653G-rs3734016C, was associated with a lower risk of cervical cancer (*P* = 0.001; OR = 0.804, 95% CI: 0.711–0.910) (Table 6). The distribution of haplotypes rs2549782G-rs2548538A-rs2248374A-rs2287988G-rs1056893T and rs2549782T-rs2548538T-rs2248374G-rs2287988A-rs1056893T in *ERAP2* (Table 7) were significantly different in the control (*P* = 0.009 and 0.003, respectively) and CIN (*P* = 0.006 and 0.009) groups compared with the cervical cancer group. The results indicated that rs2549782G-rs2548538A-rs2248374A-rs2287988G-rs1056893T may be associated with a higher risk of cervical cancer (OR = 1.592, 95% CI: 1.122–2.258) and the progression from CIN to cervical cancer (OR = 2.000, 95% CI: 1.215–3.292). Moreover, rs2549782T-rs2548538T-rs2248374G-rs2287988A-rs1056893T may be associated with a lower risk of cervical cancer (OR = 0.835, 95%CI: 0.740–0.942) and the progression from CIN to cervical cancer (OR = 0.817, 95% CI: 0.702–0.951).

**Table 6:** The distribution of the haplotypes constructed by SNPs in *ERAP1* gene

Haplotypes	Control (Freq)	CIN (Freq)	Cervical cancer (Freq)	Cervical cancer vs Control		CIN vs Control		Cervical cancer vs CIN	
				***P***-value	OR[95%CI]	***P***-value	OR[95%CI]	***P***-value	OR[95%CI]
G-C-C-C-C	646.12 (25.6%)	299.22 (26.9%)	556.04 (25.9%)	0.041	1.151 [1.006–1.316]	0.122	1.137 [0.966–1.338]	0.890	1.012 [0.855–1.197]
G-T-T-G-C	76.43 (3.0%)	37.05 (3.3%)	64.76 (3.0%)	0.612	1.091 [0.779–1.528]	0.486	1.153 [0.773–1.719]	0.794	0.947 [0.627–1.428]
C-T-T-G-C	1101.70 (43.7%)	444.48 (40.0%)	759.76 (35.4%)	0.001	0.804 [0.711–0.910]	0.260	0.918 [0.791–1.065]	0.096	0.876 [0.750–1.024]
G-C-T-C-C	196.08 (7.8%)	72.59 (6.5%)	176.09 (8.2%)	0.150	1.169 [0.945–1.447]	0.315	0.866 [0.655–1.146]	0.039	1.350 [1.015–1.795]
G-C-T-C-T	336.22 (13.3%)	142.58 (12.8%)	278.80(13.0%)	0.402	1.076 [0.906–1.278]	0.970	1.004 [0.813–1.241]	0.533	1.072 [0.862–1.334]

**Note:** The statistical significant threshold was set at *P* < 0.01 (0.05/n, *n* = 5) after Bonferroni correction

**Table 7:** The distribution of the haplotypes constructed by SNPs in *ERAP2* gene

Haplotypes	Control (Freq)	CIN (Freq)	Cervical cancer (Freq)	Cervical cancer vs Control		CIN vs Control		Cervical cancer vs CIN	
				***P***-value	OR[95%CI]	***P***-value	OR[95%CI]	***P***-value	OR[95%CI]
G-A-A-G-C	953.78 (37.8%)	411.75 (37.0%)	784.87 (36.6%)	0.219	1.080 [0.955–1.220]	0.908	0.991 [0.852–1.153]	0.336	1.079 [0.925–1.258]
G-A-A-G-T	58.71 (2.3%)	20.26 (1.8%)	72.44 (3.4%)	0.009	1.592 [1.122–2.258]	0.053	1.837 [0.983–3.434]	0.006	2.000 [1.215–3.292]
G-T-A-G-C	61.18 (2.4%)	25.61 (2.3%)	71.82 (3.3%)	0.018	1.513 [1.070–2.139]	0.638	1.130 [0.678–1.884]	0.055	1.560 [0.987–2.465]
T-T-G-A-T	1346.11 (53.3%)	586.29 (52.7%)	973.03 (45.4%)	0.003	0.835 [0.740–0.942]	0.908	1.009 [0.868–1.173]	0.009	0.817 [0.702–0.951]

**Note:** The statistical significant threshold was set at *P* < 0.012 (0.05/n, *n* = 4) after Bonferroni correction

## Discussion

The immune system is activated by MHC-peptide complexes, after which it eliminates infected and cancerous cells in various ways. The APM plays crucial roles in the initiation and development of various human diseases. As components of the APM, ERAP1 and ERAP2 are important determinants of the repertoire of peptides ultimately presented by HLA class I molecules [39–42]. Moreover, the SNPs in ERAP genes have been shown to affect the function of ERAPs by changing their peptidome or enzymatic activity [29, 30, 43]. In cervical cancer, ERAP1 and ERAP2 proteins have been reported to be highly variable, ranging from low to high expression levels [44–46]. Although there are inconsistencies among these studies, it is clear that the dysregulated expression of ERAP proteins, which may be induced by *ERAP* gene SNPs [47, 48], is associated with cervical cancer risk.

In 2007, Mehta et al. found that rs27044 in *ERAP1* was associated with cervical cancer risk. In the current study, rs27044 was found to be associated with cervical cancer risk (*P* = 0.003). The C allele of rs27044 (Q730) was found to be a protective factor for cervical cancer (OR = 0.838, 95% CI: 0.746–0.941) (Table 2), which was consistent with the results of Mehta’s study [24]. The SNP, rs27044, a non-synonymous polymorphism, leads to a Q730E substitution in the IV catalysis domain of ERAP1 [33] and may change the substrate length preferences of ERAP1 [49]. Therefore, rs27044 may play a role in cervical cancer by affecting ERAP1 function.

The SNP, rs26618, in *ERAP1* leads to an amino acid substitution (I276M) and the current study showed that the CC genotype of this SNP may be associated with an increased risk of cervical cancer (OR = 1.53; 95% CI: 1.14–2.05) compared with TT-CT genotypes (Table 4). In 2016, Guasp et al. reported that I276M (rs26618) may affect the peptidome of ERAP1 by destroying peptides with p2 Ala, unless the p1 amino acid was resistant to ERAP1 trimming [43], which indicated that rs26618 may be associated with cervical cancer. However, in a Netherlands population, Mehta et al. reported no association between rs26618 and cervical carcinoma. One of the reasons of inconsistency between our data and Mehta et al. could be the different sample sizes and statistical power. The sample size used by Mehta et al. was 251 individuals and the statistical power of rs26618 is 0.141, while 2890 individuals were enrolled in the current study and the statistical power of the same SNP is 0.621. In addition, the different population genetic background could be another reason.

In 2007, Mehta et al. reported that the C allele of rs26653 in *ERAP1* was associated with a higher cervical cancer risk in a Netherlands population [24]. In the current study, the G allele (OR = 0.829; 95% CI: 0.738–0.930) (Table 2), compared to the C allele, and the 2GG + CG genotype, compared to the CC genotype (OR = 0.82; 95% CI: 0.73–0.93) of rs26653, were associated with lower cervical cancer risk (Table 4). In 2014, Stratikos et al. and Alvarez-Navarro et al. reported that rs26653, which is a non-synonymous polymorphism resulting in a P127R substitution, may be associated with ERAP expression [18, 49], and this substitution may also affect the enzymatic activity of ERAP1 in the editing of tumour antigen peptides. This finding may explain the association between rs26653 and cervical cancer risk; however, the mechanisms need to be determined in functional studies.

In the current study, we found an association between rs2287988 in *ERAP2*, which is responsible for a synonymous polymorphism (Q563Q), and cervical cancer. The G allele may be associated with a higher risk of cervical cancer (*P* = 0.004; OR = 1.187, 95% CI: 1.057–1.332) (Table 3). Moreover, the GG-GA genotype was associated with an increased risk of cervical cancer (*P* = 0.002; OR = 1.33, 95% CI: 1.11–1.60) (Table 5). However, association studies of this SNP are rare. Previous studies have found that *ERAP2* haplotypes containing rs2287988 affect ERAP2 splicing and expression [50, 51]. Thus, additional association studies in different populations are necessary to investigate the role of this polymorphism during the initiation and development of cervical cancer.

ERAPs are markedly polymorphic and *ERAP* haplotypes whose protein products differ at multiple amino acids may affect peptide editing by ERAPs [29, 30, 52, 53]. In the current study, we also analysed haplotypes of *ERAP* SNPs in LD. The results showed that the *ERAP1* haplotype, rs27044C-rs30187T-rs26618T-rs26653G-rs3734016C and the *ERAP2* haplotypes, rs2549782T-rs2548538T-rs2248374G-rs2287988A-rs1056893T and rs2549782G-rs2548538A-rs2248374A-rs2287988G-rs1056893T may be associated with cervical cancer risk. These results indicated that SNPs in polymorphic genes may have combinatorial effects on disease susceptibility.

## Conclusion

Studies indicated that genetic factors might be correlated with cervical cancer risk [54–56], the clinical parameters of cervical cancer [57, 58] and the clinical outcome of cervical cancer [59, 60]. In the current study, we found that genetic polymorphisms in *ERAP1* and *ERAP2* genes might be associated with CIN and cervical cancer, and suggested that polymorphisms in key antigen-processing genes could affect susceptibility of cervical cancer. The strength of our study could be we investigated the association of ERAP SNPs with different stages of cervical cancer (healthy individuals, CIN and cervical cancer patients). By contrary, the limitations of our study are that we could not collect more details of the patients’ clinical parameters and had no functional verification. Association studies can only provide preliminary results for the correlation between genetic factors and cervical cancer susceptibility, the determination of the SNPs’ roles in cervical cancer requires functional studies to be resolved in the future.

## Supplementary information

**Additional file 1: Supplementary Table 1.** The SNPs selected in the current study. **Supplementary Table 2.** Inheritance model analysis of SNPs in ERAP1 gene between control and CIN groups. **Supplementary Table 3.** Inheritance model analysis of SNPs in ERAP1 gene between CIN and cervical cancer groups. **Supplementary Table 4.** Inheritance model analysis of SNPs in ERAP2 gene between control and CIN cancer groups. **Supplementary Table 5.** Inheritance model analysis of SNPs in ERAP2 gene between CIN and cervical cancer groups. **Supplementary Table 6.** The linkage disequilibrium tests of SNPs in ERAP1 gene in control group. **Supplementary Table 7.** The linkage disequilibrium tests of SNPs in ERAP2 gene in control group.

## Data Availability

The data generated during the current study are available to any scientist wishing to use them for non-commercial purpose from the corresponding author on reasonable request. However, the clinical data might be available without the privacy data of participates in the current study.

## Abbreviations

ERAP: Endoplasmic reticulum aminopeptidase
SNPs: Single nucleotide polymorphisms
APM: Antigen-processing machinery
TAPs: Transporters associated with antigen presentation
MHC: Major histocompatibility complex
HPV: Human papillomavirus
HWE: Hardy-Weinberg equilibrium
ORs: Odds ratios
CIN: Cervical intraepithelial neoplasia
SCC: squamous cell carcinoma
AC: Adenocarcinoma
CIs: confidence intervals
LD: Linkage disequilibrium
AIC: Akaike information criterion
BIC: Bayesian information criterion
