# Supplementary material for: Polymorphisms in endoplasmic reticulum aminopeptidase genes are associated with cervical cancer risk in a Chinese Han population
Source: BMC Cancer. 2020 Apr 22;20:341. doi: 10.1186/s12885-020-06832-2 (PMC7178719; doi:10.1186/s12885-020-06832-2)
Supplement: Supplementary file 1 — Additional file 1: Supplementary Table 1. The SNPs selected in the current study. Supplementary Table 2. Inheritance model analysis of SNPs in ERAP1 gene between control and CIN groups. Supplementary Table 3. Inheritance model analysis of SNPs in ERAP1 gene between CIN and cervical cancer groups. Supplementary Table 4. Inheritance model analysis of SNPs in ERAP2 gene between control and CIN cancer groups. Supplementary Table 5. Inheritance model analysis of SNPs in ERAP2 gene between CIN and cervical cancer groups. Supplementary Table 6. The linkage disequilibrium tests of SNPs in ERAP1 gene in control group. Supplementary Table 7. The linkage disequilibrium tests of SNPs in ERAP2 gene in control group. [file 12885_2020_6832_MOESM1_ESM.docx]

**Supplementary Table 1. The SNPs selected in the current study**

| **Genes** | **SNPs** | **Nucleotides change** | **Amino acids change** | **Frequency in EAS*** | **HWE in Control (Fisher’s *P*)** |
| --- | --- | --- | --- | --- | --- |
| ERAP1 | rs27037 | G>T | Intron variant | G:66%/T:34% | 0.115 |
|  | rs27044 | G>C | Q730E | G:43%/C:57% | 0.913 |
|  | rs30187 | T>C | K528R | C:55%/T:45% | 0.922 |
|  | rs26618 | T>C | I276M | T:71%/C:29% | 0.834 |
|  | rs26653 | G>C | R127P | G:52%/C:48% | 0.052 |
|  | rs3734016 | C>T | K56E | C:83%/T:17% | 0.586 |
| ERAP2 | rs2549782 | T>G | K392N | G:47%/T:53% | 0.704 |
|  | rs2548538 | T>A | P435P | T:47%/A:53% | 0.062 |
|  | rs2248374 | A>G | Intron variant | A:47%/G:53% | 0.644 |
|  | rs2287988 | G>A | Q563Q | G:47%/A:53% | 0.556 |
|  | rs1056893 | T>C | S775S | T:53%/C:47% | 0.072 |

***Note**: EAS is SNP distribution in East Asian population.

**Supplementary Table 2. Inheritance model analysis of SNPs in *ERAP1* gene between control and CIN groups**

| **SNPs** | **Models** | **Genotypes** | **Control** | **CIN** | **OR[95%CI]** | ***P*-value** | **AIC** | **BIC** |
| --- | --- | --- | --- | --- | --- | --- | --- | --- |
| rs27037 | Condominant | G/G | 359 (28.4%) | 161 (29.0%) | 1 | 0.330 | 2243.6 | 2265.6 |
|  |  | G/T | 654 (51.8%) | 270 (48.6%) | 0.92 (0.73-1.17) |  |  |  |
|  |  | T/T | 249 (19.8%) | 125 (22.5%) | 1.12 (0.84-1.49) |  |  |  |
|  | Dominant | G/G | 359 (28.5%) | 161 (29.0%) | 1 | 0.840 | 2243.8 | 2260.3 |
|  |  | G/T-T/T | 903 (71.5%) | 395 (71.0%) | 0.98 (0.78-1.22) |  |  |  |
|  | Recessive | G/G-G/T | 1013 (80.2%) | 431 (77.5%) | 1 | 0.180 | 2242.1 | 2258.6 |
|  |  | T/T | 249 (19.8%) | 125 (22.5%) | 1.18 (0.93-1.50) |  |  |  |
|  | Log-additive | --- | --- | --- | 1.05 (0.91-1.21) | 0.520 | 2243.4 | 2259.9 |
| rs27044 | Condominant | G/G | 362 (28.7%) | 175 (31.5%) | 1 | 0.460 | 2244.3 | 2266.3 |
|  |  | G/C | 626 (49.6%) | 261 (46.9%) | 0.86 (0.68-1.09) |  |  |  |
|  |  | C/C | 274 (21.7%) | 120 (21.6%) | 0.91 (0.68-1.20) |  |  |  |
|  | Dominant | G/G | 362 (28.7%) | 175 (31.5%) | 1 | 0.230 | 2242.4 | 2258.9 |
|  |  | G/C-C/C | 900 (71.3%) | 381 (68.5%) | 0.88 (0.71-1.09) |  |  |  |
|  | Recessive | G/G-G/C | 988 (78.3%) | 436 (78.4%) | 1 | 0.950 | 2243.8 | 2260.3 |
|  |  | C/C | 274 (21.7%) | 120 (21.6%) | 0.99 (0.78-1.26) |  |  |  |
|  | Log-additive | --- | --- | --- | 0.94 (0.82-1.09) | 0.420 | 2243.2 | 2259.7 |
| rs30187 | Condominant | C/C | 343 (27.2%) | 169 (30.4%) | 1 | 0.150 | 2242.0 | 2264.0 |
|  |  | C/T | 632 (50.0%) | 251 (45.1%) | 0.81 (0.64-1.02) |  |  |  |
|  |  | T/T | 287 (22.8%) | 136 (24.5%) | 0.96 (0.73-1.26) |  |  |  |
|  | Dominant | C/C | 343 (27.2%) | 169 (30.4%) | 1 | 0.160 | 2241.8 | 2258.4 |
|  |  | C/T-T/T | 919 (72.8%) | 387 (69.6%) | 0.85 (0.69-1.06) |  |  |  |
|  | Recessive | C/C-C/T | 975 (77.2%) | 420 (75.5%) | 1 | 0.430 | 2243.2 | 2259.7 |
|  |  | T/T | 287 (22.8%) | 136 (24.5%) | 1.10 (0.87-1.39) |  |  |  |
|  | Log-additive | --- | --- | --- | 0.97 (0.84-1.12) | 0.670 | 2243.7 | 2260.2 |
| rs26618 | Condominant | T/T | 678 (53.7%) | 285 (51.3%) | 1 | 0.083 | 2240.9 | 2262.9 |
|  |  | C/T | 496 (39.3%) | 215 (38.7%) | 1.03 (0.83-1.27) |  |  |  |
|  |  | C/C | 88 (7.0%) | 56 (10.1%) | 1.51 (1.05-2.18) |  |  |  |
|  | Dominant | T/T | 678 (53.7%) | 285 (51.3%) | 1 | 0.340 | 2242.9 | 2259.4 |
|  |  | C/T-C/C | 584 (46.3%) | 271 (48.7%) | 1.10 (0.90-1.35) |  |  |  |
|  | Recessive | T/T-C/T | 1174 (93.0%) | 500 (89.9%) | 1 | 0.027 | 2238.9 | 2255.4 |
|  |  | C/C | 88 (7.0%) | 56 (10.1%) | 1.50 (1.05-2.13) |  |  |  |
|  | Log-additive | --- | --- | --- | 1.14 (0.98-1.34) | 0.090 | 2241.0 | 2257.5 |
| rs26653 | Condominant | G/G | 316 (25.0%) | 124 (22.3%) | 1 | 0.230 | 2242.9 | 2264.9 |
|  |  | G/C | 665 (52.7%) | 290 (52.2%) | 1.11 (0.86-1.42) |  |  |  |
|  |  | C/C | 281 (22.3%) | 142 (25.5%) | 1.28 (0.96-1.72) |  |  |  |
|  | Dominant | C/C | 281 (22.3%) | 142 (25.5%) | 1 | 0.130 | 2241.5 | 2258.1 |
|  |  | G/G-G/C | 981 (77.7%) | 414 (74.5%) | 1.20 (0.95-1.51) |  |  |  |
|  | Recessive | G/C-C/C | 946 (75.0%) | 432 (77.7%) | 1 | 0.220 | 2242.3 | 2258.8 |
|  |  | G/G | 316 (25.0%) | 124 (22.3%) | 1.16 (0.92-1.47) |  |  |  |
|  | Log-additive | --- | --- | --- | 1.13 (0.98-1.31) | 0.090 | 2241.0 | 2257.5 |
| rs3734016 | Condominant | C/C | 921 (73.0%) | 404 (72.7%) | 1 | 0.830 | 2245.5 | 2267.5 |
|  |  | C/T | 317 (25.1%) | 139 (25.0%) | 1.00 (0.79-1.26) |  |  |  |
|  |  | T/T | 24 (1.9%) | 13 (2.3%) | 1.24 (0.63-2.47) |  |  |  |
|  | Dominant | C/C | 921 (73.0%) | 404 (72.7%) | 1 | 0.900 | 2243.8 | 2260.3 |
|  |  | C/T-T/T | 341 (27.0%) | 152 (27.3%) | 1.01 (0.81-1.27) |  |  |  |
|  | Recessive | C/C-C/T | 1238 (98.1%) | 543 (97.7%) | 1 | 0.540 | 2243.5 | 2260.0 |
|  |  | T/T | 24 (1.9%) | 13 (2.3%) | 1.24 (0.63-2.46) |  |  |  |
|  | Log-additive | --- | --- | --- | 1.03 (0.84-1.26) | 0.770 | 2243.7 | 2260.3 |

**Note:** The statistical significant threshold was set at *P*<0.0045 after Bonferroni correction.

**Supplementary Table 3. Inheritance model analysis of SNPs in *ERAP1* gene between CIN and cervical cancer groups**

| **SNPs** | **Models** | **Genotypes** | **CIN** | **Cervical cancer** | **OR[95%CI]** | ***P*-value** | **AIC** | **BIC** |
| --- | --- | --- | --- | --- | --- | --- | --- | --- |
| rs27037 | Condominant | G/G | 161 (29.0%) | 283 (26.4%) | 1 | 0.460 | 2095.4 | 2117.0 |
|  |  | G/T | 270 (48.6%) | 526 (49.1%) | 1.11 (0.87-1.41) |  |  |  |
|  |  | T/T | 125 (22.5%) | 263 (24.5%) | 1.20 (0.90-1.60) |  |  |  |
|  | Dominant | G/G | 161 (29.0%) | 283 (26.4%) | 1 | 0.270 | 2093.7 | 2109.9 |
|  |  | G/T-T/T | 395 (71.0%) | 789 (73.6%) | 1.14 (0.91-1.43) |  |  |  |
|  | Recessive | G/G-G/T | 431 (77.5%) | 809 (75.5%) | 1 | 0.350 | 2094.1 | 2110.3 |
|  |  | T/T | 125 (22.5%) | 263 (24.5%) | 1.12 (0.88-1.43) |  |  |  |
|  | Log-additive | --- | --- | --- | 1.10 (0.95-1.26) | 0.210 | 2093.4 | 2109.6 |
| rs27044 | Condominant | C/C | 175 (31.5%) | 360 (33.6%) | 1 | 0.220 | 2093.9 | 2115.5 |
|  |  | C/G | 261 (46.9%) | 520 (48.5%) | 0.97 (0.77-1.23) |  |  |  |
|  |  | G/G | 120 (21.6%) | 192 (17.9%) | 0.78 (0.59-1.05) |  |  |  |
|  | Dominant | C/C | 175 (31.5%) | 360 (33.6%) | 1 | 0.410 | 2094.3 | 2110.5 |
|  |  | C/G-G/G | 381 (68.5%) | 712 (66.4%) | 0.91 (0.73-1.14) |  |  |  |
|  | Recessive | C/C-C/G | 436 (78.4%) | 880 (82.1%) | 1 | 0.083 | 2091.9 | 2108.1 |
|  |  | G/G | 120 (21.6%) | 192 (17.9%) | 0.80 (0.62-1.03) |  |  |  |
|  | Log-additive | --- | --- | --- | 0.89 (0.77-1.03) | 0.130 | 2092.6 | 2108.8 |
| rs30187 | Condominant | C/C | 169 (30.4%) | 347 (32.4%) | 1 | 0.150 | 2093.1 | 2114.7 |
|  |  | C/T | 251 (45.1%) | 509 (47.5%) | 0.99 (0.78-1.26) |  |  |  |
|  |  | T/T | 136 (24.5%) | 216 (20.1%) | 0.78 (0.59-1.03) |  |  |  |
|  | Dominant | C/C | 169 (30.4%) | 347 (32.4%) | 1 | 0.440 | 2094.3 | 2110.5 |
|  |  | C/T-T/T | 387 (69.6%) | 725 (67.6%) | 0.92 (0.73-1.14) |  |  |  |
|  | Recessive | C/C-C/T | 420 (75.5%) | 856 (79.8%) | 1 | 0.052 | 2091.2 | 2107.3 |
|  |  | T/T | 136 (24.5%) | 216 (20.1%) | 0.78 (0.61-1.00) |  |  |  |
|  | Log-additive | --- | --- | --- | 0.89 (0.77-1.03) | 0.110 | 2092.3 | 2108.5 |
| rs26618 | Condominant | T/T | 285 (51.3%) | 546 (50.9%) | 1 | 0.990 | 2096.9 | 2118.5 |
|  |  | C/T | 215 (38.7%) | 416 (38.8%) | 1.01 (0.81-1.25) |  |  |  |
|  |  | C/C | 56 (10.1%) | 110 (10.3%) | 1.03 (0.72-1.46) |  |  |  |
|  | Dominant | T/T | 285 (51.3%) | 546 (50.9%) | 1 | 0.910 | 2094.9 | 2111.1 |
|  |  | C/T-C/C | 271 (48.7%) | 526 (49.1%) | 1.01 (0.82-1.24) |  |  |  |
|  | Recessive | T/T-C/T | 500 (89.9%) | 962 (89.7%) | 1 | 0.890 | 2094.9 | 2111.1 |
|  |  | C/C | 56 (10.1%) | 110 (10.3%) | 1.02 (0.73-1.44) |  |  |  |
|  | Log-additive | --- | --- | --- | 1.01 (0.87-1.18) | 0.890 | 2094.9 | 2111.1 |
| rs26653 | Condominant | G/G | 142 (25.5%) | 299 (27.9%) | 1 | 0.590 | 2095.9 | 2117.5 |
|  |  | C/G | 290 (52.2%) | 545 (50.8%) | 0.89 (0.70-1.14) |  |  |  |
|  |  | C/C | 124 (22.3%) | 228 (21.3%) | 0.88 (0.65-1.18) |  |  |  |
|  | Dominant | C/C | 124 (22.3%) | 228 (21.3%) | 1 | 0.660 | 2094.7 | 2110.9 |
|  |  | G/G-C/G | 432 (77.7%) | 844 (78.7%) | 0.95 (0.74-1.21) |  |  |  |
|  | Recessive | C/G-C/C | 414 (74.5%) | 773 (72.1%) | 1 | 0.310 | 2093.9 | 2110.1 |
|  |  | G/G | 142 (25.5%) | 299 (27.9%) | 0.89 (0.70-1.12) |  |  |  |
|  | Log-additive | --- | --- | --- | 0.93 (0.81-1.08) | 0.360 | 2094.1 | 2110.3 |
| rs3734016 | Condominant | C/C | 404 (72.7%) | 752 (70.2%) | 1 | 0.520 | 2095.6 | 2117.2 |
|  |  | C/T | 139 (25%) | 297 (27.7%) | 1.14 (0.90-1.45) |  |  |  |
|  |  | T/T | 13 (2.3%) | 23 (2.1%) | 0.95 (0.47-1.89) |  |  |  |
|  | Dominant | C/C | 404 (72.7%) | 752 (70.2%) | 1 | 0.310 | 2093.9 | 2110.1 |
|  |  | C/T-T/T | 152 (27.3%) | 320 (29.9%) | 1.13 (0.90-1.41) |  |  |  |
|  | Recessive | C/C-C/T | 543 (97.7%) | 1049 (97.8%) | 1 | 0.800 | 2094.9 | 2111.1 |
|  |  | T/T | 13 (2.3%) | 23 (2.1%) | 0.91 (0.46-1.82) |  |  |  |
|  | Log-additive | --- | --- | --- | 1.09 (0.89-1.34) | 0.400 | 2094.2 | 2110.4 |

**Note:** The statistical significant threshold was set at *P*<0.0045 after Bonferroni correction.

**Supplementary Table 4. Inheritance model analysis of SNPs in *ERAP2* gene between control and CIN cancer groups**

| **SNPs** | **Models** | **Genotypes** | **Control** | **CIN** | **OR[95%CI]** | ***P*-value** | **AIC** | **BIC** |
| --- | --- | --- | --- | --- | --- | --- | --- | --- |
| rs2549782 | Condominant | T/T | 395 (31.3%) | 173 (31.1%) | 1 | 0.890 | 2245.6 | 2267.6 |
|  |  | G/T | 628 (49.8%) | 282 (50.7%) | 1.03 (0.82-1.29) |  |  |  |
|  |  | G/G | 239 (18.9%) | 101 (18.2%) | 0.96 (0.72-1.29) |  |  |  |
|  | Dominant | T/T | 395 (31.3%) | 173 (31.1%) | 1 | 0.920 | 2243.8 | 2260.3 |
|  |  | G/T-G/G | 867 (68.7%) | 383 (68.9%) | 1.01 (0.81-1.25) |  |  |  |
|  | Recessive | T/T-G/T | 1023 (81.1%) | 455 (81.8%) | 1 | 0.690 | 2243.7 | 2260.2 |
|  |  | G/G | 239 (18.9%) | 101 (18.2%) | 0.95 (0.73-1.23) |  |  |  |
|  | Log-additive | --- | --- | --- | 0.99 (0.86-1.14) | 0.870 | 2243.8 | 2260.3 |
| rs2548538 | Condominant | T/T | 439 (34.8%) | 189 (34.0%) | 1 | 0.940 | 2245.7 | 2267.7 |
|  |  | A/T | 583 (46.2%) | 260 (46.8%) | 1.04 (0.83-1.30) |  |  |  |
|  |  | A/A | 240 (19.0%) | 107 (19.2%) | 1.04 (0.78-1.38) |  |  |  |
|  | Dominant | T/T | 439 (34.8%) | 189 (34.0%) | 1 | 0.730 | 2243.7 | 2260.2 |
|  |  | A/T-A/A | 823 (65.2%) | 367 (66.0%) | 1.04 (0.84-1.28) |  |  |  |
|  | Recessive | T/T-A/T | 1022 (81.0%) | 449 (80.8%) | 1 | 0.920 | 2243.8 | 2260.3 |
|  |  | A/A | 240 (19.0%) | 107 (19.2%) | 1.01 (0.79-1.31) |  |  |  |
|  | Log-additive | --- | --- | --- | 1.02 (0.89-1.17) | 0.780 | 2243.8 | 2260.3 |
| rs2248374 | Condominant | G/G | 382 (30.3%) | 169 (30.4%) | 1 | 0.680 | 2245.1 | 2267.1 |
|  |  | A/G | 632 (50.0%) | 287 (51.6%) | 1.03 (0.82-1.29) |  |  |  |
|  |  | A/A | 248 (19.7%) | 100 (18.0%) | 0.91 (0.68-1.22) |  |  |  |
|  | Dominant | G/G | 382 (30.3%) | 169 (30.4%) | 1 | 0.970 | 2243.8 | 2260.3 |
|  |  | A/G-A/A | 880 (69.7%) | 387 (69.6%) | 1.00 (0.80-1.24) |  |  |  |
|  | Recessive | G/G-A/G | 1014 (80.3%) | 456 (82.0%) | 1 | 0.400 | 2243.1 | 2259.6 |
|  |  | A/A | 248 (19.7%) | 100 (18.0%) | 0.90 (0.69-1.16) |  |  |  |
|  | Log-additive | --- | --- | --- | 0.96 (0.83-1.11) | 0.620 | 2243.6 | 2260.1 |
| rs2287988 | Condominant | A/A | 387 (30.7%) | 167 (30.0%) | 1 | 0.730 | 2245.2 | 2267.2 |
|  |  | A/G | 633 (50.1%) | 289 (52.0%) | 1.06 (0.84-1.33) |  |  |  |
|  |  | G/G | 242 (19.2%) | 100 (18.0%) | 0.96 (0.71-1.29) |  |  |  |
|  | Dominant | A/A | 387 (30.7%) | 167 (30%) | 1 | 0.770 | 2243.7 | 2260.3 |
|  |  | A/G-G/G | 875 (69.3%) | 389 (70%) | 1.03 (0.83-1.28) |  |  |  |
|  | Recessive | A/A-A/G | 1020 (80.8%) | 456 (82%) | 1 | 0.540 | 2243.5 | 2260.0 |
|  |  | G/G | 242 (19.2%) | 100 (18%) | 0.92 (0.71-1.19) |  |  |  |
|  | Log-additive | --- | --- | --- | 0.99 (0.86-1.14) | 0.880 | 2243.8 | 2260.3 |
| rs1056983 | Condominant | T/T | 439 (34.8%) | 195 (35.1%) | 1 | 0.990 | 2245.8 | 2267.8 |
|  |  | C/T | 584 (46.3%) | 257 (46.2%) | 0.99 (0.79-1.24) |  |  |  |
|  |  | C/C | 239 (18.9%) | 104 (18.7%) | 0.98 (0.74-1.30) |  |  |  |
|  | Dominant | T/T | 439 (34.8%) | 195 (35.1%) | 1 | 0.920 | 2243.8 | 2260.3 |
|  |  | C/T-C/C | 823 (65.2%) | 361 (64.9%) | 0.99 (0.80-1.22) |  |  |  |
|  | Recessive | T/T-C/T | 1023 (81.1%) | 452 (81.3%) | 1 | 0.900 | 2243.8 | 2260.3 |
|  |  | C/C | 239 (18.9%) | 104 (18.7%) | 0.98 (0.76-1.27) |  |  |  |
|  | Log-additive | --- | --- | --- | 0.99 (0.86-1.14) | 0.890 | 2243.8 | 2260.3 |

**Note:** The statistical significant threshold was set at *P*<0.0045 after Bonferroni correction.

**Supplementary Table 5. Inheritance model analysis of SNPs in *ERAP2* gene between CIN and cervical cancer groups**

| **SNPs** | **Models** | **Genotypes** | **CIN** | **Cervical cancer** | **OR[95%CI]** | ***P*-value** | **AIC** | **BIC** |
| --- | --- | --- | --- | --- | --- | --- | --- | --- |
| rs2549782 | Condominant | T/T | 173 (31.1%) | 304 (27.8%) | 1 | 0.340 | 2112.6 | 2134.3 |
|  |  | G/T | 282 (50.7%) | 571 (52.2%) | 1.15 (0.91-1.45) |  |  |  |
|  |  | G/G | 101 (18.2%) | 219 (20.0%) | 1.23 (0.91-1.67) |  |  |  |
|  | Dominant | T/T | 173 (31.1%) | 304 (27.8%) | 1 | 0.170 | 2110.9 | 2127.1 |
|  |  | G/T-G/G | 383 (68.9%) | 790 (72.2%) | 1.17 (0.94-1.46) |  |  |  |
|  | Recessive | T/T-G/T | 455 (81.8%) | 875 (80.0%) | 1 | 0.350 | 2111.9 | 2128.2 |
|  |  | G/G | 101 (18.2%) | 219 (20.0%) | 1.13 (0.87-1.47) |  |  |  |
|  | Log-additive | --- | --- | --- | 1.12 (0.96-1.29) | **0.150** | **2110.7** | **2126.9** |
| rs2548538 | Condominant | T/T | 189 (34.0%) | 351 (32.1%) | 1 | 0.680 | 2114.0 | 2135.7 |
|  |  | A/T | 260 (46.8%) | 517 (47.3%) | 1.06 (0.84-1.34) |  |  |  |
|  |  | A/A | 107 (19.2%) | 226 (20.7%) | 1.14 (0.85-1.52) |  |  |  |
|  | Dominant | T/T | 189 (34.0%) | 351 (32.1%) | 1 | 0.460 | 2112.2 | 2128.5 |
|  |  | A/T-A/A | 367 (66.0%) | 743 (67.9%) | 1.09 (0.87-1.35) |  |  |  |
|  | Recessive | T/T-A/T | 449 (80.8%) | 868 (79.3%) | 1 | 0.490 | 2112.3 | 2128.5 |
|  |  | A/A | 107 (19.2%) | 226 (20.7%) | 1.10 (0.85-1.42) |  |  |  |
|  | Log-additive | --- | --- | --- | 1.07 (0.92-1.23) | 0.380 | 2112.0 | 2128.3 |
| rs2248374 | Condominant | G/G | 169 (30.4%) | 282 (25.8%) | 1 | 0.077 | 2109.7 | 2131.3 |
|  |  | A/G | 287 (51.6%) | 578 (52.8%) | 1.20 (0.95-1.53) |  |  |  |
|  |  | A/A | 100 (18.0%) | 234 (21.4%) | 1.41 (1.04-1.90) |  |  |  |
|  | Dominant | G/G | 169 (30.4%) | 282 (25.8%) | 1 | 0.050 | 2108.9 | 2125.2 |
|  |  | A/G-A/A | 387 (69.6%) | 812 (74.2%) | 1.26 (1.00-1.57) |  |  |  |
|  | Recessive | G/G-A/G | 456 (82.0%) | 860 (78.6%) | 1 | 0.094 | 2110.0 | 2126.2 |
|  |  | A/A | 100 (18.0%) | 234 (21.4%) | 1.25 (0.96-1.62) |  |  |  |
|  | Log-additive | --- | --- | --- | 1.19 (1.02-1.38) | 0.024 | 2107.7 | 2123.9 |
| rs2287988 | Condominant | A/A | 167 (30.0%) | 279 (25.5%) | 1 | 0.073 | 2109.6 | 2131.2 |
|  |  | A/G | 289 (52.0%) | 578 (52.8%) | 1.19 (0.94-1.51) |  |  |  |
|  |  | G/G | 100 (18.0%) | 237 (21.7%) | 1.42 (1.05-1.92) |  |  |  |
|  | Dominant | A/A | 167 (30.0%) | 279 (25.5%) | 1 | 0.056 | 2109.1 | 2125.4 |
|  |  | A/G-G/G | 389 (70.0%) | 815 (74.5%) | 1.25 (1.00-1.57) |  |  |  |
|  | Recessive | A/A-A/G | 456 (82.0%) | 857 (78.3%) | 1 | 0.074 | 2109.6 | 2125.8 |
|  |  | G/G | 100 (18.0%) | 237 (21.7%) | 1.27 (0.98-1.64) |  |  |  |
|  | Log-additive | --- | --- | --- | 1.19 (1.03-1.38) | 0.022 | 2107.6 | 2123.8 |
| rs1056983 | Condominant | T/T | 195 (35.1%) | 373 (34.1%) | 1 | 0.920 | 2114.6 | 2136.3 |
|  |  | C/T | 257 (46.2%) | 512 (46.8%) | 1.04 (0.83-1.31) |  |  |  |
|  |  | C/C | 104 (18.7%) | 209 (19.1%) | 1.05 (0.79-1.41) |  |  |  |
|  | Dominant | T/T | 195 (35.1%) | 373 (34.1%) | 1 | 0.700 | 2112.6 | 2128.9 |
|  |  | C/T-C/C | 361 (64.9%) | 721 (65.9%) | 1.04 (0.84-1.29) |  |  |  |
|  | Recessive | T/T-C/T | 452 (81.3%) | 885 (80.9%) | 1 | 0.820 | 2112.7 | 2129.0 |
|  |  | C/C | 104 (18.7%) | 209 (19.1%) | 1.03 (0.79-1.34) |  |  |  |
|  | Log-additive | --- | --- | --- | 1.03 (0.89-1.19) | 0.700 | 2112.6 | 2128.9 |

**Note:** The statistical significant threshold was set at *P*<0.0045 after Bonferroni correction.

**Supplementary Table 6 The linkage disequilibrium tests of SNPs in *ERAP1* gene in control group**

| **D’/r^2^** | **rs27044** | **rs30187** | **rs26618** | **rs26653** | **rs3734016** |
| --- | --- | --- | --- | --- | --- |
| **rs27037** | 0.619/0.370 | 0.603/0.333 | 0.556/0.094 | 0.616/0.302 | 0.810/0.093 |
| **rs27044** | - | 0.919/0.803 | 0.978/0.302 | 0.940/0.728 | 0.941/0.130 |
| **rs30187** | - | - | 1.000/0.332 | 0.970/0.815 | 0.944/0.138 |
| **rs26618** | - | - | - | 0.977/0.366 | 0.895/0.049 |
| **rs26653** | - | - | - | - | 0.934/0.156 |

**Supplementary Table 7 The linkage disequilibrium tests of SNPs in *ERAP2* gene in control group**

| **D’/r^2^** | **rs2548538** | **rs2248374** | **rs2287988** | **rs1056893** |
| --- | --- | --- | --- | --- |
| **rs2549782** | 0.943/0.829 | 0.993/0.953 | 0.972/0.928 | 0.952/0.843 |
| **rs2548538** | - | 0.947/0.807 | 0.942/0.814 | 0.878/0.769 |
| **rs2248374** | - | - | 0.962/0.910 | 0.958/0.824 |
| **rs2287988** | - | - | - | 0.946/0.818 |
